# Persistent viral activity, cytokine storm, and lung fibrosis in a case of severe COVID‐19

**DOI:** 10.1002/ctm2.224

**Published:** 2020-11-03

**Authors:** Gang Xu, Yang Liu, Mingfeng Liao, Jizhou Gou, Xin Wang, Jing Yuan, Weilong Liu, Guangde Zhou, Shuye Zhang, Lei Liu, Zheng Zhang

**Affiliations:** ^1^ Institute for Hepatology, National Clinical Research Center for Infectious Disease Shenzhen Third People's Hospital, the Second Affiliated Hospital, School of Medicine, Southern University of Science and Technology Shenzhen China; ^2^ Department for Pathology Shenzhen Third People's Hospital, the Second Affiliated Hospital, School of Medicine, Southern University of Science and Technology Shenzhen China; ^3^ Department for Infectious Diseases Shenzhen Third People's Hospital, the Second Affiliated Hospital, School of Medicine, Southern University of Science and Technology Shenzhen China; ^4^ Shanghai Public Health Clinical Center Fudan University Shanghai China

To the Editor:

The pandemic of coronavirus disease 2019 (COVID‐19) caused by SARS‐CoV‐2 has posed a great threat to public health.^1^ Although the majority of COVID‐19 patients have mild diseases, some develop acute respiratory distress syndrome.^2^, ^3^, ^4^ Understanding the mechanism of COVID‐19 severity is a prerequisite for the development of effective vaccines or therapies.

Here, we report a particular case with severe COVID‐19 to reveal some mechanistic insights. A 66‐year‐old man with pre‐existing hypertension, developed symptoms, including fever, dry cough, myalgia, and joint pain, and traveled from Wuhan to Shenzhen in China. His infection was diagnosed on day 4 postadmission, based on his airway sample being positive for SARS‐CoV‐2. The clinical condition of this patient progressively deteriorated after hospitalization, and he eventually succumbed despite of all medical intervention attempts to save his life, including experimental antiviral therapy, extracorporeal membrane oxygenation, convalescent plasma transfusion, and lung transplantation (Table 1). Dynamic monitoring of the SARS‐CoV‐2 showed viral presence in bronchoalveolar lavage fluid (BALF) or the upper airway samples for more than 23 days. Although the SARS‐CoV‐2 is undetectable 17 days later in multiple samplings (Table 1), we identified the viral RNA in his lung tissues by qPCR (Figure 1A). Viral presence was further confirmed by in situ RNA hybridization, and some were colocalized with the *ACE2* RNA probes (Figure 1B). Moreover, viral nucleocapsid protein (NP) and spike proteins in the lung were detected by immunoblotting (Figure 1C). Finally, coronavirus‐like particles were identified by electron microscopy (Figure 1D). It is intriguing to observe the SARS‐CoV‐2 persistence in the lung, while the upper respiratory samples remained virus negative, suggesting a viral reservoir and ongoing viral activity deep in the SARS‐CoV‐2‐infected lung. Therefore, effective antiviral treatment would be a necessary part of the therapy for patients with severe COVID‐19 even if SARS‐CoV‐2 RNA is undetectable in airway samples.

**TABLE 1:** The timeline of this patient's clinical course from his admission to death

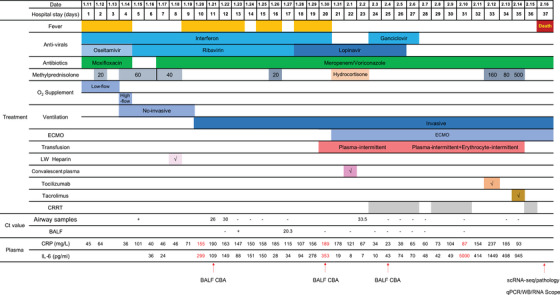

**FIGURE 1:**
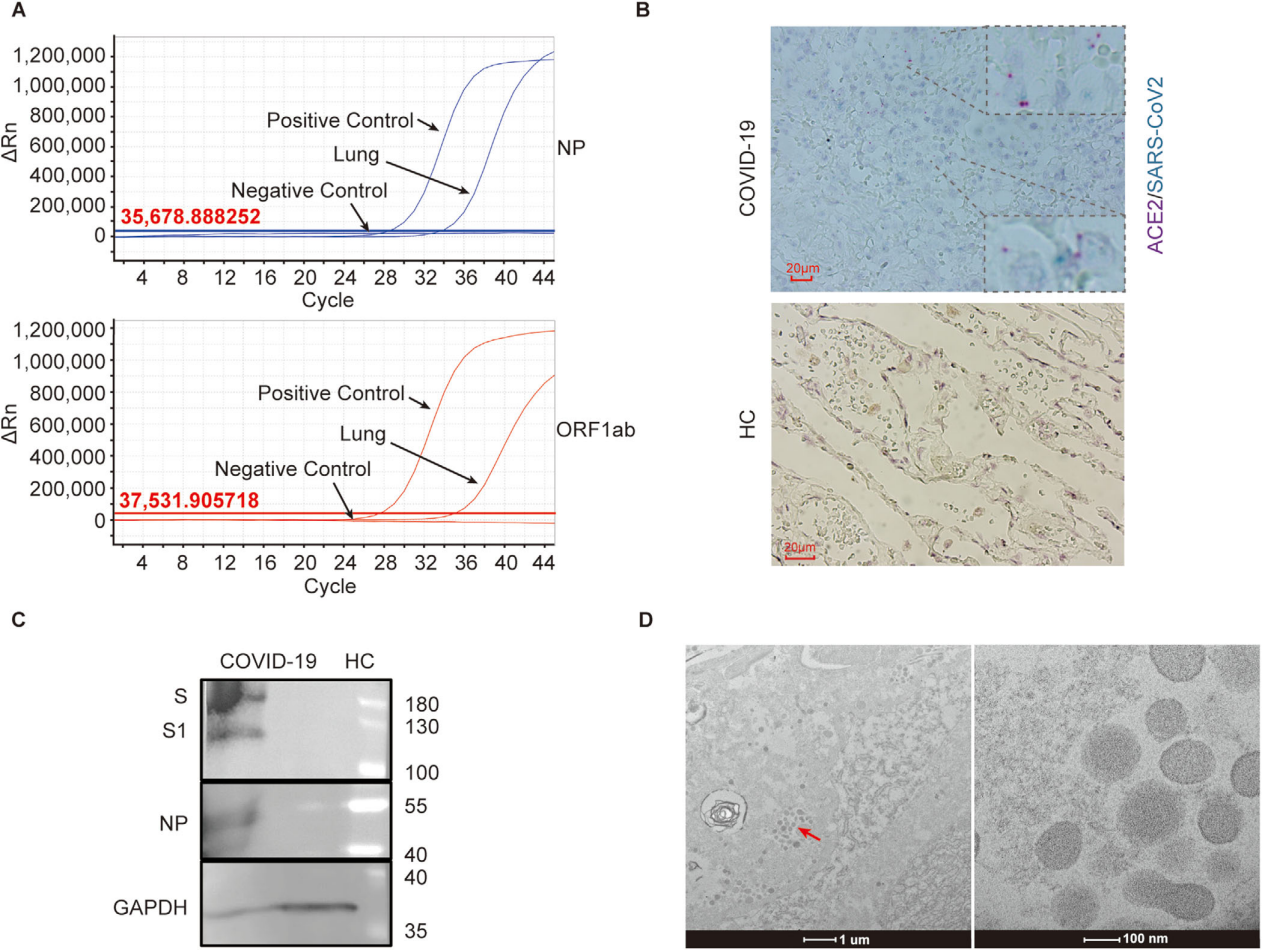
Persistent of SARS‐COV‐2 in the patient's lung. A, The presence of SARS‐CoV‐2 RNA was examined by qPCR with two probes targeting ORF1ab and nucleocapsid protein (NP), respectively. B, One cross‐section of paraffin‐embedded lung tissue from COVID‐19 patient and healthy control was hybridized by SARS‐COV‐2‐specific chromogenic probe (green) and ACE2‐specific probe (red). C, Lung tissue lysates of a control donor and this patient were analyzed by immunoblot using anti‐Spike and anti‐NP antibody. D, Thin‐layer electron microscopic photograph shows the presence of coronavirus particles (around 100 nm in diameter) in the patient's lung. Right panel is the magnification of the marked section of the left panel

To further understand the pathogenesis, the patient's lung was taken for histopathological examination. Significant pathological changes included extensive alveolar damage with alveolar epithelium disorganization, necrosis, shedding, atresia, and abundant mucus with bleeding in the dilated small bronchi, bronchioles, and terminal bronchioles (Figure S1A). Unlike previous reports on pathological changes in COVID‐19 patients,^5^, ^6^ obvious lung fibrosis was observed in this patient as indicated by Sirius Red, Masson staining, and collagen IHC (Figure 2A), suggesting that severe COVID‐19 caused pulmonary fibrosis in this case.

**FIGURE 2:**
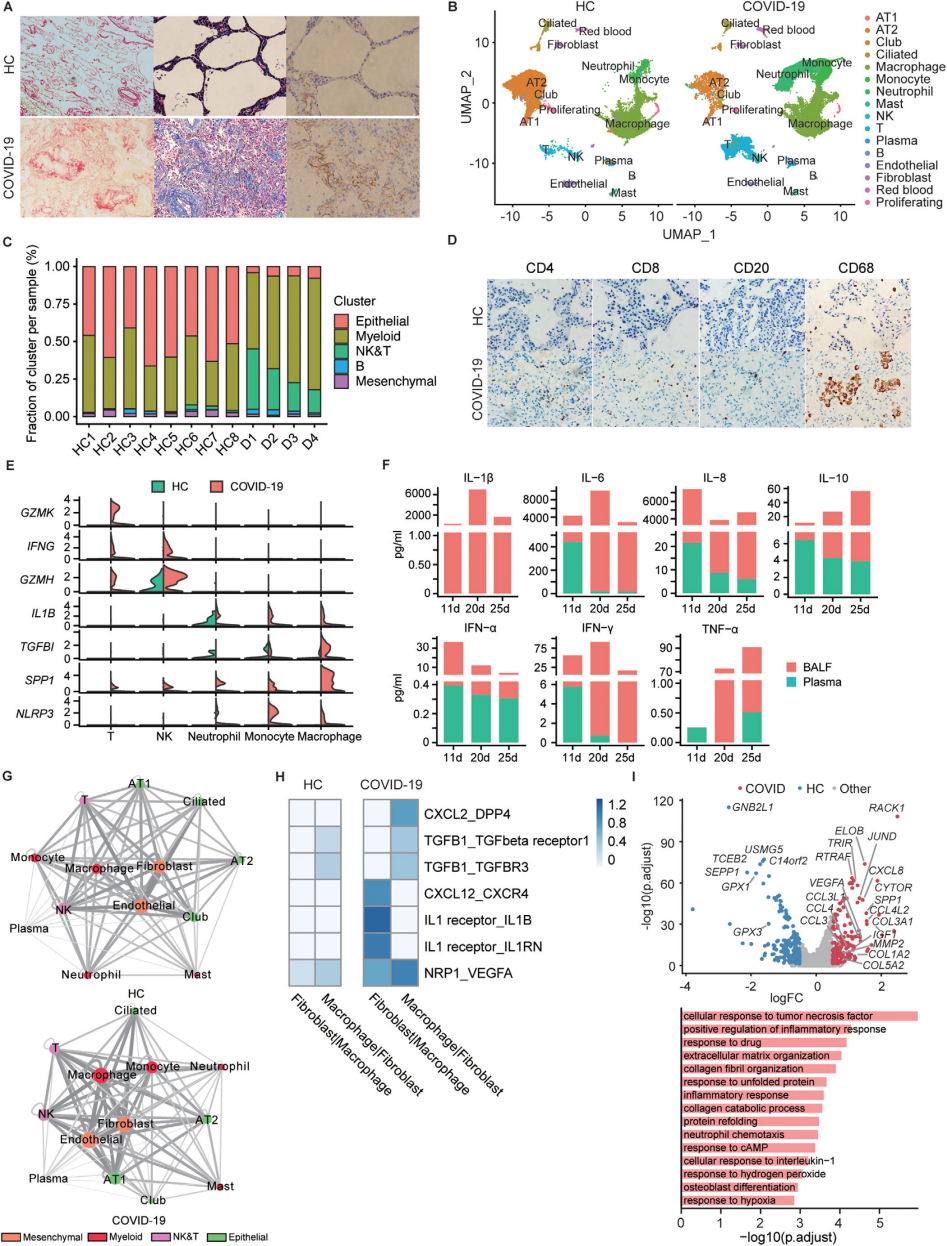
Pathological manifestations and single‐cell transcriptomes of the patient's lung. A, Sirius Red staining (left), Masson staining (middle), and immunohistochemical staining of type IV collagen (right) in healthy and COVID‐19 patient's lung revealed extensive lung fibrosis in the case. B, The uniform manifold approximation and projection (UMAP) presentation of lung single‐cell transcriptomes showing the clusters of major cell types. The first 50 principle components were loaded as input to UMAP for visualization and clustered cells using a graph‐based method. C, Bar plot showing the proportion of major cell types in each sample. D1, D2, D3, and D4 are samples collected from four different regions of the patient's lung. D, Immunohistochemical staining of patient's lung tissues with CD4, CD8, CD20, and CD68 antibody. E, The violin plots display the expression of selected immune genes across different immune subsets in healthy versus patient's lung. F, The levels of selected cytokines and chemokines in serial bronchoalveolar lavage fluid (BALF) and plasma samples (collected at day 11, 20, and 25 during the hospitalization) are displayed. G, Cell‐cell interaction networks estimated in HC (left) and COVID‐19 patient (right). The thickness of the lines represents the number of interactions between the cell types. H, Selected ligand‐receptor pairs between fibroblasts and macrophages in control versus patient's lung. The color represents total mean of the individual partner average expression values in the corresponding interacting pairs of cell types. I, Volcano plot (top) showing differentially expressed genes of lung fibroblasts in patients and healthy controls. A gene is considered significant with adjust *P* < 0.05 and logFC > 0.5. (Bottom) Selected GO terms of the significantly upregulated genes of the patient's fibroblasts

To dissect the lung microenvironment, four different regions of the lungs were taken for scRNA‐seq study. We integrated 32 191 cells from the patient and 81 447 cells from eight controls from a publicly available source (GSE122960)^7^ for analysis. Major cell types were identified by known markers, including epithelial cells (AT1, AT2, club, and ciliated cells), NK and T lymphocytes (NK, CD4, and CD8 T), mesenchymal cells (fibroblast and endothelial), myeloid cells (macrophage, monocyte, and neutrophil), B lymphocytes, and erythrocytes (Figure 2B and Figure S1B). Indeed, we observed a substantial reduction of parenchymal cells in the patient's lungs, especially epithelial cells. In contrast, the pool of immune cells, including T cells, B cells, and myeloid cells, was markedly expanded (Figure 2C). Extensive immune cell infiltration was also revealed by immunohistochemistry staining of CD4, CD8, CD20, and CD68 (Figure 2D). Gene expression analysis showed that many of those infiltrating immune cell subsets were highly activated. Compared with control, effector molecules *GZMK*, GZMH, *IFNG* were highly expressed in T cells, and *IL1B*, *NLRP3, SPP1*, and *TGFΒ1* were highly expressed in lung macrophages from the patients (Figure 2E). In addition, monitoring of multiple inflammatory factors showed that the patient was undergoing an inflammatory cytokine storm. The levels of inflammatory cytokines in BALF were much higher (Figure 2F). Long‐term monitoring of IL6 and CRP in plasma showed that these two inflammatory factors fluctuated but maintained at high levels despite of receiving glucocorticoid treatment (Table 1). Thus, effort to relieve the cytokine storm is likely a key component of successful treatment for severe COVID‐19 patients. This is consistent with the fact that recent clinical trials targeting the inflammation are showing promising results.^8^

To explore the potential molecular mechanism underlying lung fibrosis in severe COVID‐19, we mapped receptor‐ligand pairs among different cell subsets using the scRNA‐seq data to construct a putative cell‐cell interaction network. The center of interaction network in controls were endothelial cells and fibroblasts, likely a requirement for normal lung homeostasis, whereas macrophages and fibroblasts moved to the center in the patient (Figure 2G), suggesting that macrophage‐derived signals might play major roles in shaping the patient's lung microenvironment. The analysis further showed that *CXCL12* highly expressed in the patient's fibroblasts might enhance the recruitment of multiple immune populations (expressing *CXCR4*), including macrophages, T cells, and NK cells, while the patient's macrophages produced more *IL1B* and *TGFB* to modulate fibroblast functions in this patient (Figure 2H). Indeed, the patient's fibroblasts showed activated phenotype and increased expression of extracellular matrix molecules, such as collagens and matrix metalloproteinases (Figure 2I). Taken together, our data highlight the cross‐talk between macrophages and fibroblasts through well‐known IL‐1β and TGF‐β signaling pathways^9^ as an underlying drive for the lung fibrosis in severe COVID‐19.

In summary, this case reveals pathological mechanism of severe COVID‐19, including pulmonary SARS‐CoV‐2 persistence, robust inflammatory responses, and extensive lung fibrosis, which likely constitute the basis for the grave clinical outcomes and difficult treatment of patients with COVID‐19.

## FUNDING INFORMATION

Natural Science Foundation of Guangdong Province, Grant Number: 2019A1515011072; National Natural Science Foundation of China, Grant Number: 81700540

## CONFLICT OF INTEREST

The authors declare that there is no conflict of interest.

## AUTHOR CONTRIBUTIONS

Guangde Zhou, Shuye Zhang, Lei Liu, and Zheng Zhang designed this study and wrote the paper. Gang Xu performed this study and wrote the paper. Mingfeng Liao, Jizhou Gou, and Weilong Liu processed the lung tissue and performed immunohistochemistry experiments. Yang Liu performed bioinformatics analysis. Jing Yuan provided patient's clinical information. Gang Xu and Xin Wang constructed the single cell library. All authors approved the final manuscript.

## Supporting information

Supporting Information

Figure S1

## References

[1] Zhu N , Zhang D , Wang W , et al. A novel coronavirus from patients with pneumonia in China, 2019. N Engl J Med. 2020;382:727‐733. 31978945 10.1056/NEJMoa2001017 PMC7092803

[2] Huang C , Wang Y , Li X , et al. Clinical features of patients infected with 2019 novel coronavirus in Wuhan, China. Lancet. 2020;395:497‐506. 31986264 10.1016/S0140-6736(20)30183-5 PMC7159299

[3] Chan JF , Yuan S , Kok KH , et al. A familial cluster of pneumonia associated with the 2019 novel coronavirus indicating person‐to‐person transmission: a study of a family cluster. Lancet. 2020;395:514‐523. 31986261 10.1016/S0140-6736(20)30154-9 PMC7159286

[4] Wang D , Hu B , Hu C , et al. Clinical characteristics of 138 hospitalized patients with 2019 novel coronavirus‐infected pneumonia in Wuhan, China. JAMA. 2020;323(11):1061‐1069. 32031570 10.1001/jama.2020.1585 PMC7042881

[5] Xu Z , Shi L , Wang Y , et al. Pathological findings of COVID‐19 associated with acute respiratory distress syndrome. Lancet Respir Med. 2020;8:420‐422. 32085846 10.1016/S2213-2600(20)30076-X PMC7164771

[6] Tian S , Hu W , Niu L , Liu H , Xu H , Xiao SY . Pulmonary pathology of early‐phase 2019 novel coronavirus (COVID‐19) pneumonia in two patients with lung cancer. J Thorac Oncol. 2020;15:700‐704. 32114094 10.1016/j.jtho.2020.02.010 PMC7128866

[7] Reyfman PA , Walter JM , Joshi N , et al. Single‐cell transcriptomic analysis of human lung provides insights into the pathobiology of pulmonary fibrosis. Am J Respir Crit Care Med. 2019;199:1517‐1536. 30554520 10.1164/rccm.201712-2410OC PMC6580683

[8] Guo C , Li B , Ma H , et al. Single‐cell analysis of two severe COVID‐19 patients reveals a monocyte‐associated and tocilizumab‐responding cytokine storm. Nat Commun. 2020;11(1):3924‐. 32764665 10.1038/s41467-020-17834-w PMC7413381

[9] Thomas BJ , Kan OK , Loveland KL , Elias JA , Bardin PG . In the shadow of fibrosis: innate immune suppression mediated by transforming growth factor‐beta. Am J Respir Cell Mol Biol. 2016;55:759‐766. 27603223 10.1165/rcmb.2016-0248PS

