# Supplementary material for: Persistent viral activity, cytokine storm, and lung fibrosis in a case of severe COVID‐19
Source: Clin Transl Med. 2020 Nov 3;10(7):e224. doi: 10.1002/ctm2.224 (PMC7607551; doi:10.1002/ctm2.224)
Supplement: Supplementary file 1 — Supporting Information [file CTM2-10-e224-s002.docx]

Persistent viral activity, cytokine storm and lung fibrosis in a case of severe COVID-19

Gang Xu,^1^^*^ Yang Liu,^1*^ Mingfeng Liao,^1*^ Jizhou Gou,^2*^ Xin Wang,^1^ Jing Yuan,^3^ Weilong Liu,^1^ Guangde Zhou,^2^**^†^** Shuye Zhang,^4^**^†^** Lei Liu,^1^**^†^** Zheng Zhang^1^**^†^**

^1^Institute for Hepatology, National Clinical Research Center for Infectious Disease, Shenzhen Third People’s Hospital, the Second Affiliated Hospital, School of Medicine, Southern University of Science and Technology, Shenzhen, China

^2^Department for Pathology, Shenzhen Third People’s Hospital, the Second Affiliated Hospital, School of Medicine, Southern University of Science and Technology, Shenzhen, China

^3^Department for Infectious Diseases, Shenzhen Third People’s Hospital, the Second Affiliated Hospital, School of Medicine, Southern University of Science and Technology, Shenzhen, China

^4^Shanghai Public Health Clinical Center, Fudan University, Shanghai 201508, China.

**^*^These authors contributed equally.**

**^†^Correspondence:**

Zheng Zhang, PhD, MD. Institute for Hepatology, Shenzhen 3^rd^ People’s Hospital, Shenzhen, Guangdong Province 518100, China; Phone: 86-755-81238983; Fax: 86-755-81238983; Email: [zhangzheng1975@aliyun.com](mailto:zhangzheng1975@aliyun.com), Lei Liu, MD. Shenzhen 3^rd^ People’s Hospital, Shenzhen, Guangdong Province 518100, China; Email: [liulei3322@aliyun.com](mailto:liulei3322@aliyun.com), Shuye Zhang, PhD. Shanghai Public Health Clinical Center, Fudan University, Shanghai, China; Email: [zhangshuye@shphc.org.cn](mailto:zhangshuye@shphc.org.cn), and Guangde Zhou, PhD. Department for Pathology, Shenzhen Third People’s Hospital, Shenzhen 518112, Guangdong Province, China; Email: guangdez@sina.com

**Methods**

**Ethics statement**: This study was reviewed and approved by the Medical Ethical Committee of Shenzhen Third People’s Hospital (2020-143). Written informed consent was obtained from the patient.

**Lung tissue processing**

Lung tissue was homogenized by mincing with scissors into smaller pieces (~0.5 mm2 per piece). The tissue was transferred into 10 ml of enzyme mix consisting of 1 mg/ml collagenase type IV (STEMCELL, 07426) and DNase I (Sigma, D5025) for mild enzymatic digestion for 1 h at 37°C with constant shaking. Five ml of PBS supplemented with 10% FBS was added to stop digestion and dissociated cells in suspension were passed through a 70 μm strainer and centrifuged at 300 g for 5 min at 4°C. Red blood cells were lysed using Ammonium-Chloride-Potassium (ACK, Thermo, A1049201), and finally cells were re-suspended in Dulbecco's phosphate-buffered saline (DPBS, Thermo, 14190250) at the concentration of 2, 000/μl for scRNA-Seq.

**qPCR**

Throat swab, Nasal swab, Anal swab, Sputum, BALF and lung tissue were collected from the patients at various time points after hospitalization. Total RNA was extracted with QIAamp RNA Viral Kit (Qiagen, Heiden, Germany), and quantitative reverse transcription polymerase chain reaction (qRT-PCR) was performed with a commercial kit specific for SARS-CoV-2 detection (GeneoDX Co., Ltd., Shanghai, China), which used the probes and primers recommended by the China CDC to target NP and ORF1ab. The samples were positive if the Ct value was lower than 37 and negative if the repeated testing remained negative (Ct > 37).

**Immunoblot**

Healthy lung tissue was from a donor of lung transplant with IRB approval. Tissues were homogenized in lysis buffer containing Protease Inhibitor Cocktail (Sigma, P8340). After homogenizing and centrifuging, the samples were boiled in 1% SDS loading buffer containing DTT, resolved by 9% SDS-PAGE and transferred to PVDF membranes following standard protocols. The following antibodies were used: anti- SARS-CoV Nucleoprotein (Sina Biological, 40143-T62), anti-SARS-CoV2-RBD (isolated from a COVID-19 patient, P2A-1A10), anti-GAPDH (TransGen Biotech, HC301-01)

**RNAscope**

In situ hybridization studies were performed on formalin fixed paraffin-embedded (FFPE) 4-μm thick tissue sections using ACE2 and SARS-COV-2 specific commercially obtained RNAscope Target Probes (ACD, 848561 and 848151-C2). RNAscope® 2.5 HD Duplex Reagent Kit (ACD, 322430) was used per manufacturer’s recommendations. Negative control probe (ACD, 320751) targeting DapB (Bacillus subtilis strain) and positive probes (ACD, 321641) of PPAB (red) and PORL2A (green) were used to confirm the success of the assay. FFPE lung tissue from a lung transplant donor was used as the control. All images were captured using the Zeiss Axio Scope. A1 microscope /HD Camera through OPLENIC Software.

**Electron microscopy**

Lung tissues were dissected and incubated in a fixative consisting of 2.5% glutaraldehyde for 2h at 4 °C and then fixed in 1% OsO4 for 2h at 4°C. Pre-embedding staining was performed overnight at 4°C in the dark using 0.5% uranyl acetate. Samples were dehydrated in ethanol series (25%, 50%, 75%, 95%, and 100%). For plastic embedding, samples were incubated in 3:1 ethanol: resin, 1:1 ethanol: resin, and 1:3 ethanol: resin for 40 min for each, then kept overnight in 100% resin. Samples were put into fresh resin and further permeated for 3 h at room temperature. Resin polymerization was done at 60°C for at least 48 h. Resin blocks were cut with an ultra-microtome (Leica) and serial sections of 100-nm thickness were collected on formvar copper grid (KYKY) and visualized under an electron microscope (FEI, Talos L120C) with a voltage acceleration of 120 kV.

**Immunohistochemistry**

The procedure involved deparaffinizing the slices in dimethylbenzene, oxidizing the slices with 1% periodate for 30 min and rinsing with distilled water. The sections were immersed into hexamine silver for 35 min (water bath 60°C) and decolorized by 0.1% gold chloride (100 μl). The tissues were fixed with 3% sodium thiosulfate for 1 min, replenished with the bouin's solution (37°C water bath for 4 h) and the sections were washed with distilled water for 5 min, stained t by Mayer hematoxylin and put into hot water (45°C) for 30 s. The specimens were stained in Masson solution (100 μl) for 30 min. The sections were differentiated by 1% phosphomolybdic acid (100 μl). Phosphomolybdic acid was removed and the sections were added with 1% aniline blue (100 μl), rinsed with distilled water, and added with 1% acetic acid. The sections were finally dehydrated by 95% and 100% alcohol (10 seconds and 1 minute, respectively) and sealed.

**Masson staining**

Deparaffinize the slices in dimethylbenzene, oxidize the slices with 1% periodate for 30 min and rinse with distilled water. Immerse the sections into hexamine silver for 35 min (water bath 60°C). Decolorize the sections by 0.1% gold chloride (100 μl). Fix the tissues with 3% sodium thiosulfate for 1 min, replenish by the bouin's solution (37°C water bath for 4 h) and wash the sections by distilled water for 5 min. Stain the sections by Mayer hematoxylin and put into hot water (45°C) for 30 s. Dye the specimens by Masson solution (100 μl) for 30 min. Differentiate the sections by 1% phosphomolybdic acid (100 μl). Remove phosphomolybdic acid and add the sections with 1% aniline blue (100 μl). Rinse the sections with distilled water, add 1% acetic acid. At last, dehydrate the sections by 95% and 100% alcohol (10 seconds and 1 minute, respectively) and seal.

**Cytokines measurement by CBA**

Cytokines including IL-1β, IL-6, IL-8, IL-10, IFN-γ, IFN- α, TNF-α in BALF and plasma were detected according to the instruction (Uni-medica, Shenzhen, China, Cat. No. 503022). In brief, after centrifugation, 25 μl BALF supernatant or plasma was taken. Twenty-five μl Sonicate Beads, and 25 μl of Detection Antibodies were added and mixed well by shaking at 500 rpm for 2 hours at room temperature. Then 25 μl of SA-PE was added and constantly shaken at 500 rpm for 30 minutes. The data were obtained by flow cytometry (Canto II, BD) and were analyzed using LEGENDplex v8.0 (VigeneTech Inc).

**ScRNA-Seq library construction based on the 10X genomics platform**

The scRNA-Seq libraries were prepared with Chromium Single Cell 3ʹ Reagent Kits v3 (10x Genomics; PN-1000075, PN-1000073 and PN-120262) following the user guide provided. Briefly, Gel bead in Emulsion (GEM) were generated by combining barcoded Gel Beads, a Master Mix containing 20, 000 cells, and Partitioning Oil onto Chromium Chip B. Reverse transcription (RT) takes place inside each GEM, after which cDNAs are pooled for amplification and library construction in bulk. Finished library molecules consisted of Illumina adapters and sample indices, allowing pooling and sequencing of multiple libraries on a next-generation short read sequencer.

**Healthy Control Data**

The lung scRNA-seq data from the healthy controls was acquired from the Gene Expression Omnibus (GEO) database under the series number GSE122960, which contained data of lung tissue from eight lung transplant donors generated using 3' V2 chemistry kit on Chromium Single cell controller (10xGenomics). Filtered feature-barcode matrix was used in the following analysis.

**ScRNA-seq data alignment and sample aggregating**

The Cell Ranger Software Suite (Version 3.1.0) was used to perform sample de-multiplexing, barcode processing and single-cell 3’ UMI counting with human GRCh38 as the reference genome. Specifically, splicing-aware aligner STAR was used in FASTQs alignment. Cell barcodes were then determined based on distribution of UMI count automatically. Following criteria were then applied to each cell of all 4 disease samples and 8 healthy controls; i.e., gene number between 200 and 6, 000, UMI count above 1, 000 and mitochondrial gene percentage below 0.1. Finally, filtered gene-barcode matrix of all samples was integrated with Seurat v3 to remove batch effect across different samples. In parameter settings, the first 50 dimensions of CCA and PCA were used.

**Dimensionality reduction and clustering**

The filtered gene-barcode matrix was first normalized using ‘LogNormalize’ methods in Seurat v3 with default parameters. The top 2, 000 variable genes were then identified using ‘vst’ method in Seurat FindVariableFeatures function. Variables nCount_RNA and percent.mito were regressed out in the scaling step and principal component analysis (PCA) was performed using the top 2, 000 variable genes. Then Uniform Manifold Approximation and Projection (UMAP) was then performed on the top 50 principal components for visualizing the cells. Meanwhile, graph-based clustering was performed on the PCA-reduced data for clustering analysis with Seurat v3, with the resolution being set at 1.0 to get a finer result.

**Differential gene expression analysis for clusters**

MAST in Seurat v3 was used to perform differential gene expression analysis. For each cluster, differentially-expressed genes (DEGs) were generated relative to all of the other cells.

**Gene functional annotation**

For DEGs of lung fibroblasts in patients and healthy controls, Gene ontology (GO) analysis was performed with DAVID 6.8.

**Cell-cell communication**

Cell–cell communication mediated by ligand–receptor complexes is critical to coordinating diverse biological processes, such as development, differentiation and inflammation. CellphoneDB is a repository of ligands, receptors and their interactions and was used to infer cell-cell communications of different cell types with default parameters. Network visualization was performed using Cytoscape (version 3.7.2). The network layout was set to force-directed layout. In the network, the thickness of the lines represents the number of interactions between the cell types. In the heatmap of figure 2H, the color represents the total mean of the individual partner average expression values in the corresponding interacting pairs of cell types.

**Code availability**

The source code and software pipeline to reproduce our analyses can be assessed upon request.


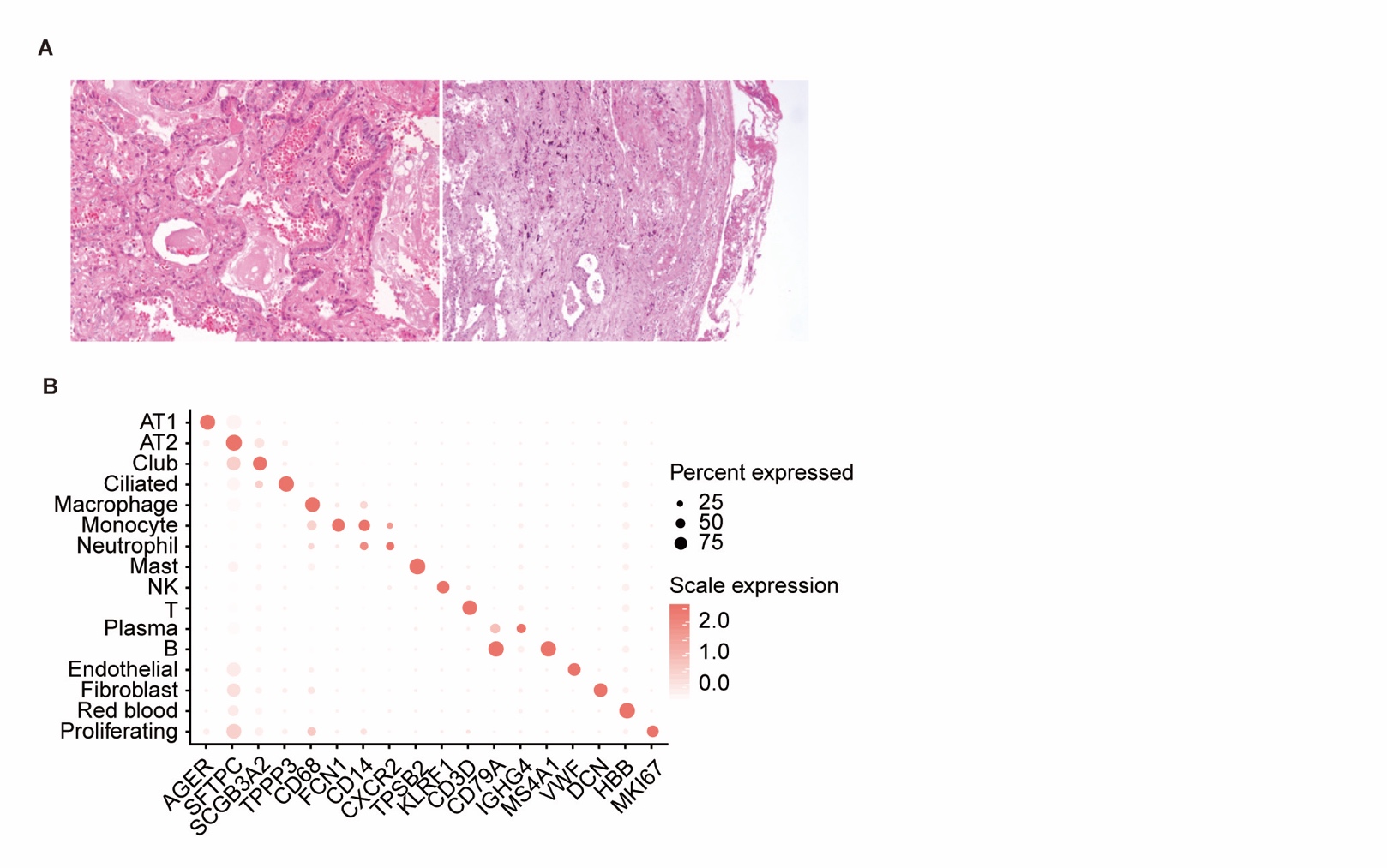


Supplementary Figure S1 HE staining of the patient’s lung and specific markers of the various cell types.

(A) HE staining of the patient’s lung sections show the loss and necrosis of epithelial cells, deposition of massive mucus in pulmonary alveoli and presence of ground glass lesions.

(B) The heatmap showing the hallmark genes specifically expressed by different cell cluster.
