# Supplementary figures and images for: Persistent viral activity, cytokine storm, and lung fibrosis in a case of severe COVID‐19
Source: Clin Transl Med. 2020 Nov 3;10(7):e224. doi: 10.1002/ctm2.224 (PMC7607551; doi:10.1002/ctm2.224)

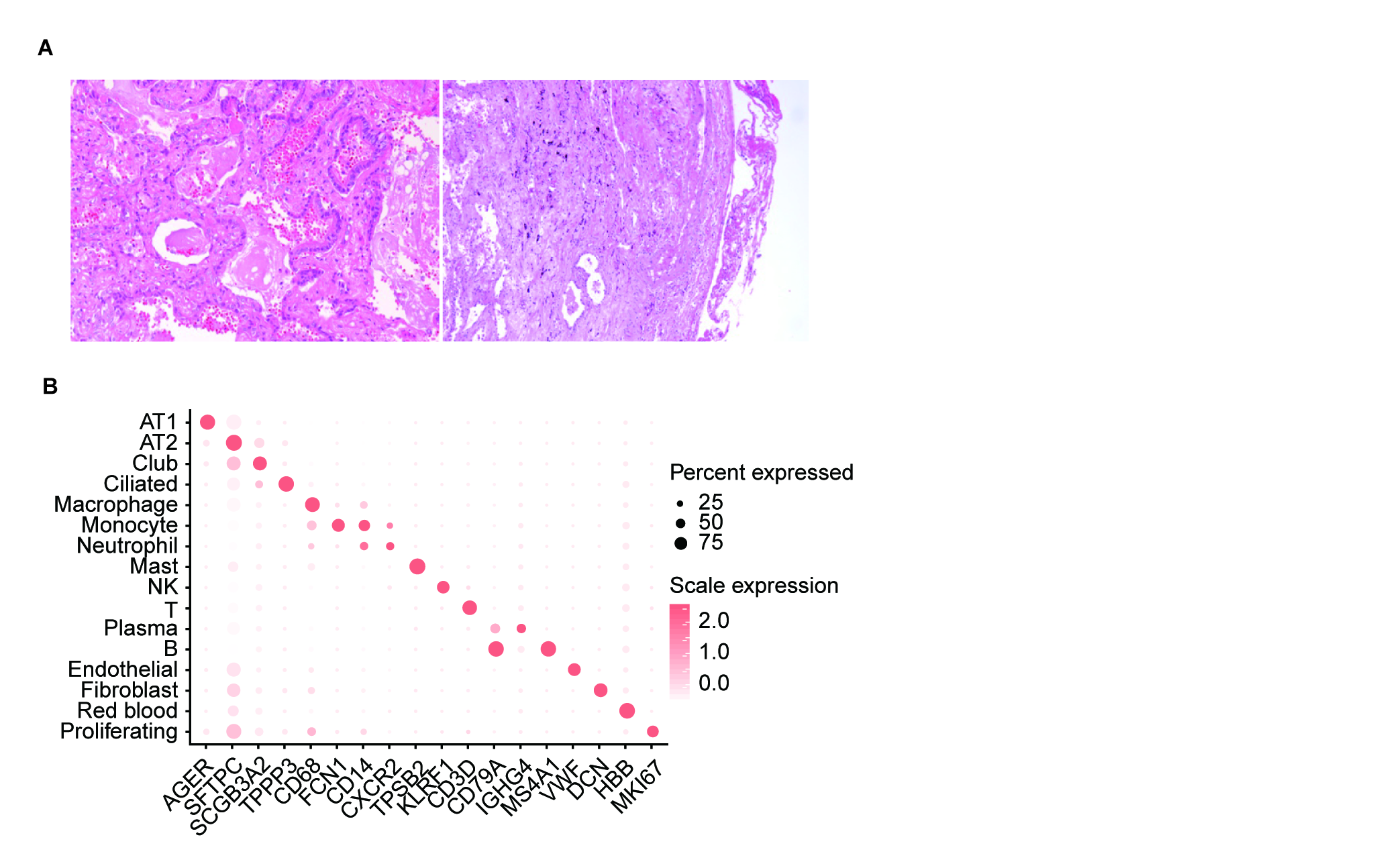

Supplement: Supplementary file 2 — Figure S1 [file CTM2-10-e224-s001.tif]
